# Effect of glutathione reductase knockdown in response to UVB-induced oxidative stress in human lung adenocarcinoma

**DOI:** 10.1186/1477-5956-12-2

**Published:** 2014-01-09

**Authors:** Hsiu-Chuan Chou, Hong-Lin Chan

**Affiliations:** 1 Department of Applied Science, National Hsinchu University of Education, Hsinchu, Taiwan; 2 Institute of Bioinformatics and Structural Biology & Department of Medical Sciences, National Tsing Hua University, Hsinchu, Taiwan

**Keywords:** Glutathione reductase, Proteomics, Redox-proteomics, 2D-DIGE, MALDI-TOF MS

## Abstract

**Background:**

Glutathione reductase (GR) plays a critical role in the maintenance of physiological redox status in cells. However, the comprehensive investigations of GR-modulated oxidative stress have not been reported.

**Methods:**

In the present study, we cultured a human lung adenocarcinoma line CL1-0 and its GR-knockdown derivative CL1-0ΔGR to evaluate their differential responses to UVB-irradiation.

**Results:**

We identified 18 proteins that showed significant changes under UVB-irradiation in CL1-0ΔGR cells rather than in CL1-0 cells. Several proteins involving protein folding, metabolism, protein biosynthesis and redox regulation showed significant changes in expression.

**Conclusions:**

In summary, the current study used a comprehensive lung adenocarcinoma-based proteomic approach for the identification of GR-modulated protein expression in response to UVB-irradiation. To our knowledge, this is the first global proteomic analysis to investigate the role of GR under UVB-irradiation in mammalian cell model.

## Introduction

Oxidative and reductive modifications of proteins are one of the primary mechanisms to regulate the biological functions of these proteins. In fact, numerous chemical moieties have been described to be probable targets of reactive oxygen species (ROS) in cells. One of these potential targets is the free thiol group of cysteine residues which are potent nucleophilic target and are able to undertake numerous redox-induced protein modifications under physiological conditions. Oxidative modifications of free thiol groups are not only to form the disulfide bond between cysteine residues, also to modify the free thiol groups into the sulfenic acid, sulfinic acid and sulfonic acid depending on the relative oxidative capacity of the oxidant [1]. Importantly, oxidation of free thiol groups to sulfinic and sulfonic acids is irreversible reaction under physiologic conditions and might induce loss of biological functions of proteins [2,3].

ROS can modify various biological molecules chemically and induce cell injury. Recent researches demonstrated that UV-induced reactive oxidant species (ROS) which can activate growth factor receptors, cytokine receptors as well as intracellular signaling molecules [4-6]. In all kinds of UV irradiation, UVB is primarily considered to modulate cell physiology [7-9]. Accordingly, cells have evolved a number of mechanisms to defense oxidative damage by means of the generation of intracellular antioxidants, the expression of redox-regulated enzymes as well as the maintenance of high concentrations of ROS scavengers such as glutathione (GS). The sulfhydryl form GS (GSH) can reduce oxidized thiol groups or hydrogen peroxide via glutathione peroxidase to form disulfide-linked GS (GSSG). The GSH can be regenerated from GSSG through glutathione reductase (GR) with the reducing source from cellular nicotinamide adenine dinucleotide phosphate (NADPH). Thus, GR plays a critical role in the maintenance of correct redox status in cells [10].

GR is an important anti-oxidative enzyme in many organisms including bacteria, yeasts and animals but especially, in plants as a key enzyme in the ascorbate-glutathione cycle. The ascorbate-glutathione cycle is one of the crucial and efficient anti-oxidant systems for removal of ROS as well as the maintenance of cellular redox balance in plant [11-14]. In addition, the ascorbate-glutathione cycle involves the recycling pathway of ascorbate and glutathione and plays essential role in maintaining the reduced forms of ascorbate and glutathione in plant cell to protect plants against oxidative stresses [15]. Hence, GR is not only a key protein to modulate cellular oxidative stress but closely relates to global crop yields.

In current research, we have studied the differential protein expression between CL1-0 and its GR-knockdown derivative CL1-0ΔGR in response to UVB-irradiation. To examine the differentially expressed levels of cellular proteins, a quantitative proteomics-based approach was performed using lysine labeling-2D-DIGE combining MALDI-TOF MS analysis [16-20] to obtain a panel of proteins found to be differentially altered with/without GR depletion in protein abundance.

## Materials and methods

### Chemicals and reagents

Generic chemicals were purchased from Sigma-Aldrich (St. Louis, USA), and the reagents for 2D-DIGE were purchased from GE Healthcare (Uppsala, Sweden). All the chemicals and biochemicals used in this study were analytical grade. All western blot assay used primary antibodies were purchased from Genetex (Hsinchu, Taiwan) and anti-rabbit secondary antibodies were purchased from GE Healthcare (Uppsala, Sweden).

### Cell lines and cell cultures

CL1-0, human lung adenocarcinoma cells, used in this studies were transfected with pLKO.1 vector in the present of puromycin (0.8 μg/ml) and cultured in DMEM medium supplemented with 10% fetal bovine serum, L-glutamine (2 mM), streptomycin (100 μg/mL), penicillin (100 IU/mL) (all from Gibco-Invitrogen Corp., UK). The glutathione reductase knockdown strain, CL1-0ΔGR, was selected from CL1-0 cells transfected with the pLKO.1-shGR vector in the present of puromycin (0.8 μg/ml) as described previously [21]. All cells were incubated at 37°C in a humidified atmosphere containing 5% CO_2_.

### GR activity assays

GR activity was measured using the glutathione reductase assay kit from Sigma-Aldrich, which is a colorimetric assay monitoring the reduction of DTNB. 100 μg of CL1-0 and CL1-0ΔGR cell extracts was used directly in this assay. One unit of enzyme activity caused the reduction of 1 μmol of DTNB to TNB at 25°Cat pH 7.5.

### Oxidized protein analysis

The amount of oxidized proteins in CL1-0 and CL1-0ΔGR cells were determined by using an OxyElisa Oxidized Protein Quantitation Kit from Millipore according to the manufacturer’s instructions.

### GSH/GSSG measurement

Reduced and oxidized Glutathione ratios of CL1-0 and CL1-0ΔGR cells were measured by using the GSH/GSSG-Glo assay kit from Promega according to the manufacturer’s protocol.

### UVB treatment

CL1-0 and CL1-0ΔGR cells were cultured in culture medium at about 80% confluence treated with various UVB doses in each experiment by machine CL1000 UV cross-linker fitted with 5 × 8 W 302 nm dual bipin discharge type tubes with the lid off after removing medium and washing with PBS twice. Cells were then grown at defined time points for functional assays or 2D-DIGE analysis.

### Immunoblotting analysis

Immunoblotting analysis was used to validate the differential abundance of mass spectrometry identified proteins and glutathione reductase level in CL1-0 and CL1-0ΔGR. The detailed experimental procedures have been described in our previous study [22-24]. All primary antibodies used for expression validation were purchased from Genetex (Hsinchu, Taiwan).

### Assay for endogenous reactive oxygen species using DCFH-DA

The detailed experimental procedures have been described in our previous study [22,25-27]. Briefly, CL1-0 and CL1-0ΔGR cells (10,000 cells/well) were irradiated with the indicated doses of UVB. After two washes with PBS, cells were treated with 10 μM of 2, 7-dichlorofluorescin diacetate (DCFH-DA; Molecular Probes) at 37°Cfor 20 min, and subsequently washed with PBS. Fluorescence was recorded at an excitation wavelength 485 nm and emission wavelength at 530 nm.

### MTT cell viability assay

The detail MTT procedure has been described in our previous publication [28]. Briefly, CL1-0 and CL1-0ΔGR cells growing exponentially were trypsinized and seeded at a density of 5,000 cells per well into 96-well plates. After washing with PBS three times, the CL1-0 and CL1-0ΔGR cells were transiently irradiated with indicated doses of UVB or left untreated followed by a further incubation at 37°C for 24 h to carry out MTT assay. The detail MTT procedure has been described in our previous publication [28].

### 2D-DIGE, gel image analysis, protein staining, in-gel digestion and MALDI-TOF MS analysis

The detailed experimental procedures have been described in our previous study [17,27,28]. Briefly, CL1-0 and CL1-0ΔGR cells in normal growth medium at ~80% confluence were used for proteomic analysis. CL1-0 and CL1-0ΔGR cells with UVB-irradiation or left without irradiation were lysed with 2-DE lysis buffer. Before performing 2D-DIGE, protein samples were labeled with N-hydroxy succinimidyl ester-derivatives of the cyanine dyes Cy2, Cy3 and Cy5 according to the previous publications [17]. 150 μg of protein sample was minimally labeled with 375 pmol of either Cy3 or Cy5 for comparison on the same 2-DE. In contrast, to facilitate image matching and cross-gel statistical comparison, a pool of all samples was also prepared and labeled with Cy2 at a molar ratio of 2.5 pmol Cy2 per μg of protein as an internal standard for all gels. Thus, the triplicate samples and the internal standard could be run and quantify on multiple 2-DE. Afterward, the fluorescence 2-DE were scanned directly between the low fluorescent glass plates using an Ettan DIGE Imager and gel analysis was performed using DeCyder 2-D Differential Analysis Software v 7.0 (GE Healthcare) to co-detect, normalize and quantify the protein features in the images. Features detected from non-protein sources (e.g. dust particles and dirty backgrounds) were filtered out. Spots displaying a ≧ 1.3 average-fold increase or decrease in CL1-0ΔGR protein abundance but a ≦ 1.2 average-fold increase or decrease in CL1-0 protein abundance with a p-value < 0.05 in response to UVB irradiation were selected for protein identification.

### Protein staining, in-gel digestion and MALDI-TOF MS analysis

Colloidal coomassie blue G-250 staining was used to visualize CyDye-labeled protein features in 2-DE followed by excised interested post-stained gel pieces for MALDI-TOF MS identification. The detailed procedures for protein staining, in-gel digestion, MALDI-TOF MS analysis and the algorithm used for data processing were described in our previous publication [17]. The spectrometer was calibrated with a peptide calibration standard (Bruker Daltonics), and internal calibration was performed using trypsin autolysis peaks at *m*/*z* 842.51 and *m/z* 2211.10. Peaks in the mass range of m/z 800–3000 were used to generate a peptide mass fingerprint that was searched against the Swiss-Prot/TrEMBL database (released on November 2011) with 533 049 entries using Mascot software v2.3.02 (Matrix Science, London, UK). The following parameters were used for the search: *Homo sapiens*; tryptic digest with a maximum of 1 missed cleavage; carbamidomethylation of cysteine, partial protein N-terminal acetylation, partial methionine oxidation and partial modification of glutamine to pyroglutamate and a mass tolerance of 50 ppm. Identification was accepted based on significant MASCOT Mowse scores (*p* < 0.05), matched peptide sequence coverage higher than 15%, spectrum annotation and observed versus expected molecular weight and p*I* on 2-DE.

## Results

### GR knockdown-induced alterations in GR expression, GR activity, protein carbonylation and intracellular GSH/GSSG ratio in CL1-0 and CL1-0ΔGR cells

**Figure 1:**
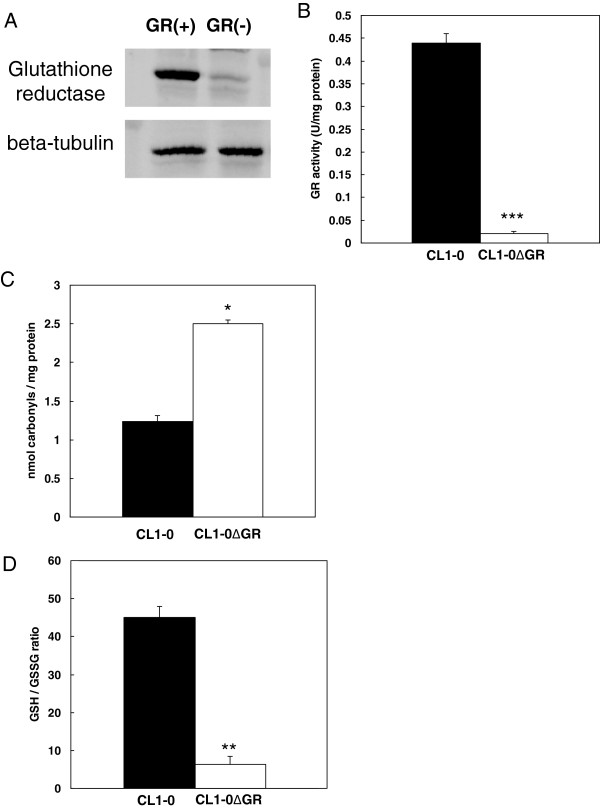
**GR knockdown-induced alterations in GR expression, GR activity, protein carbonylation and intracellular GSH/GSSG ratio in CL1-0 and CL1-0ΔGR cells. (A)** CL1-0 cells and CL1-0∆GR cells grown overnight and the expression of glutathione reductase in these 2 cell lines were monitored by immunoblotting. Beta-tubulin was used as loading control in this study. **(B)** GR activity assays of CL1-0 cells and CL1-0∆GR cells were performed. Activity is reported as units per milligram of protein in the total cell extract. One unit of enzyme activity is equal to 1 μmol DTNB reduced/min at 25°Cat pH 7.5. **(C)** Oxidative carbonylation of proteins in CL1-0 cells and CL1-0∆GR cells were measured by colorimetric-based ELISA analysis. **(D)** Reduced and oxidized glutathione ratio in CL1-0 cells and CL1-0∆GR cells were measured by luminescence-based ELISA analysis. Values are the average of 3 independent measurements +/- the standard deviation (p < 0.05*, p < 0.01 ** and p < 0.001 ***).

In order to investigate the differential protein expression between CL1-0 and its GR-knockdown derivative CL1-0ΔGR in response to UVB-irradiation, the CL1-0ΔGR lung cancer cells were selected from CL1-0 cells transfected with the GR shRNA in puromycin containing medium. The immunoblotting results indicated that CL1-0ΔGR showed a significant down-regulation in GR level in comparison with the GR levels in CL1-0 implying the CL1-0 and CL1-0ΔGR cells are appropriate to be used as a GR-depletion cell model to study GR-modulated cellular protein expression in response to UVB-irradiation (Figure 1A). Subsequent characterization of GR knockdown-induced alterations in GR activity, protein carbonylation and intracellular GSH/GSSG ratio in CL1-0 and CL1-0ΔGR cells demonstrated that GR knockdown resulted in significantly reducing of GR activity and GSH/GSSG ratio in CL1-0ΔGR cells. In contrast, GR knockdown caused obviously increasing of protein oxidation such as protein carbonylation in CL1-0ΔGR cells (Figure 1B-D).

### Effect of UVB irradiation on cell viability in CL1-0 and CL1-0ΔGR cells

**Figure 2:**
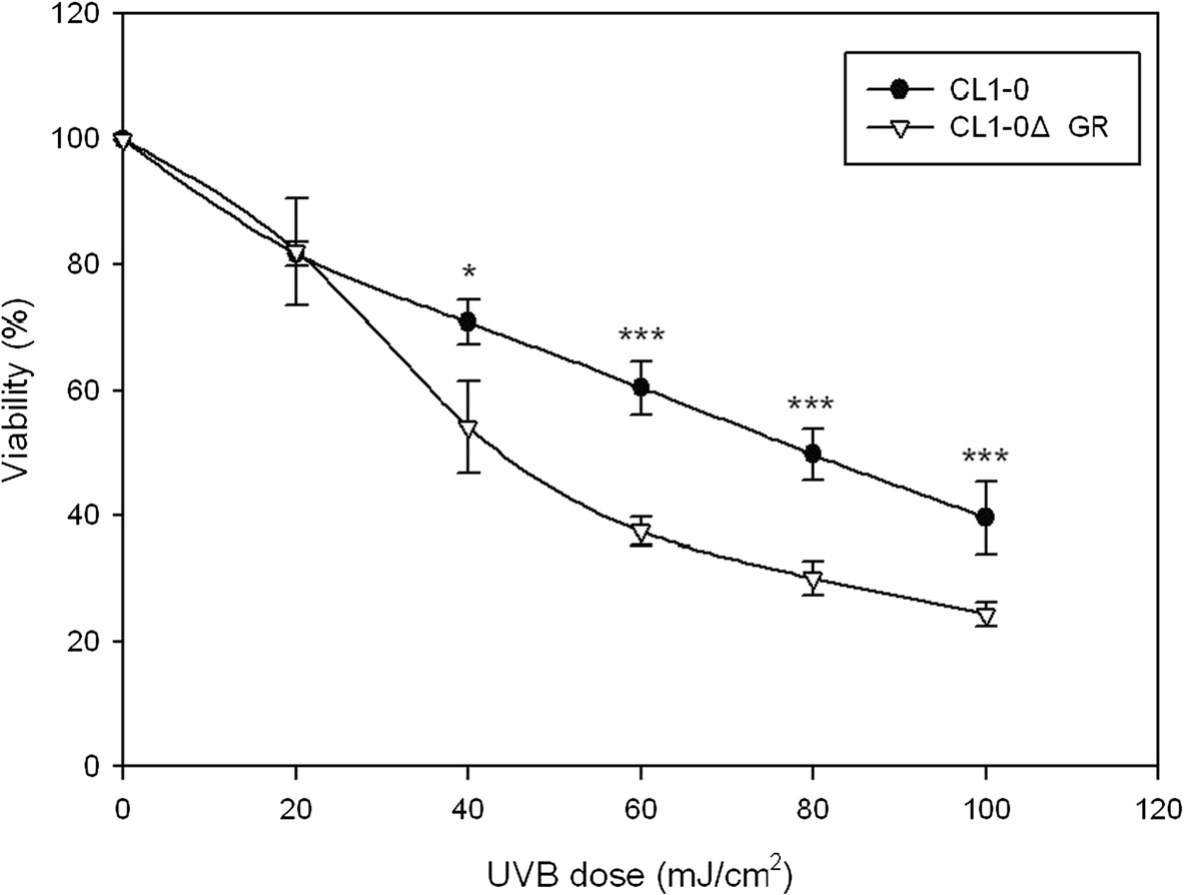
**UVB-induced loss of cell viability in CL1-0 and CL1-0ΔGR cells.** MTT-based viability assays were performed where 5,000 CL1-0 and CL1-0∆GR cells were plated into 96-well plates in medium containing 10% FBS. After 24 h incubation, the cells were irradiated with the indicated doses of UVB for another 24 h. Cells were incubated with MTT and then DMSO added and the plates shaken for 20 min followed by measurement of the absorbance at 540 nm. Values were normalized against untreated samples and are the average of 4 independent measurements +/- the standard deviation (p < 0.05*, p < 0.001 ***).

To study the effect of UVB irradiation on cell viability in CL1-0 and CL1-0ΔGR cells, the two cells were exposed to 302 nm dual bipin discharge type UVB tubes with UVB doses at 0, 20, 40, 60, 80, 100 mJ/cm^2^. As expected from the dosage used, irradiation of CL1-0 and CL1-0ΔGR cells to UVB was shown to result in a dose-dependent loss of cell viability (Figure 2). At UVB doses of 45 mJ/cm^2^ and 80 mJ/cm^2^, a significant loss of cell viability (50%) for CL1-0ΔGR cells and CL1-0 cells were detected in 24 h incubation, respectively (Figure 2).

### Effect of UVB irradiation on ROS production in CL1-0 and CL1-0ΔGR cells

**Figure 3:**
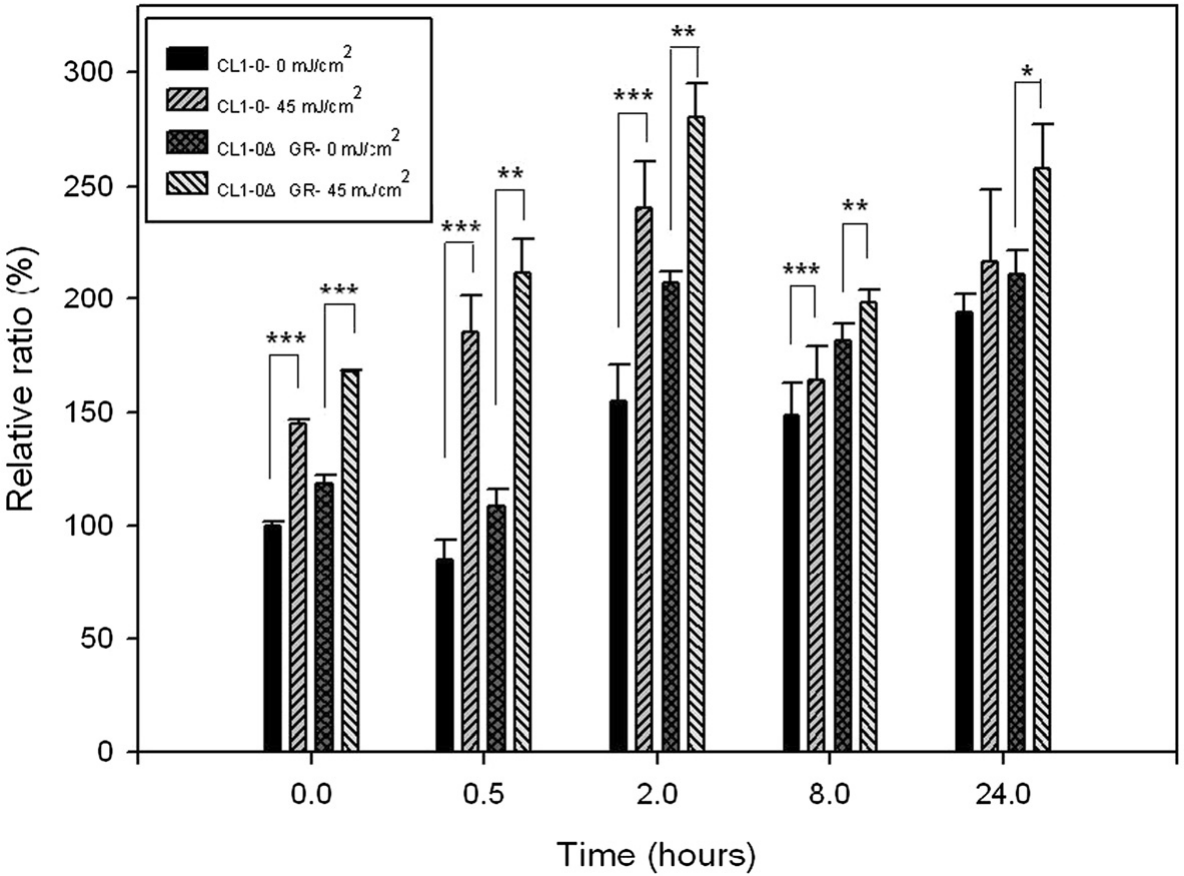
**Effect of UVB on CL1-0 and CL1-0ΔGR ROS production.** Microtiter plate-based DCFH analysis of intracellular ROS production assays were performed where CL1-0 and CL1-0∆GR cells (50,000 cells / well) were plated into 24-well plates. After 24 h, the cells were treated with 45 mJ/cm^2^ of UVB. Cells were further incubated for indicated periods before treated with 10 μM of DCFH-DA at 37°Cfor 20 min and the fluorescence was recorded at excitation and emission wavelengths of 485 nm and 530 nm, respectively. Data were represented as mean ± SD (p < 0.05*, p < 0.01**, p < 0.001 ***).

GR has long been recognized to reduce intracellular ROS level via reducing GSSG into GSH. The depletion of GR might result in ROS accumulation and mediate modifications on bio-molecules such as lipids, DNA and proteins in cells. In order to study the effect of UVB irradiation on the generation of intracellular ROS in CL1-0 and CL1-0ΔGR cells, DCFH-DA analysis has been applied to monitor intracellular ROS level alteration. These two cell lines were exposed to 302 nm UVB for 45 mJ/cm^2^ followed by incubated for 0, 0.5, 2, 8 and 24 h before treated with 10 mM of DCFH-DA. As expected from the UVB applied, irradiation of CL1-0 and CL1-0ΔGR cells to UVB was shown to result in increased intracellular ROS in both cells with the maximal ROS production at 2 h post-UVB irradiation (Figure 3). Further investigation demonstrated that CL1-0ΔGR cells generated higher levels of ROS in comparison with the ROS production in CL1-0 cells in response to UVB irradiation at all of indicated post-UVB irradiation time courses (Figure 3) implying GR plays important roles in maintaining cellular redox-homeostasis in response to UVB irradiation.

### 2D-DIGE and MALDI-TOF MS analysis of UVB irradiation-induced differential protein expression between CL1-0 and CL1-0ΔGR cells

**Figure 4:**
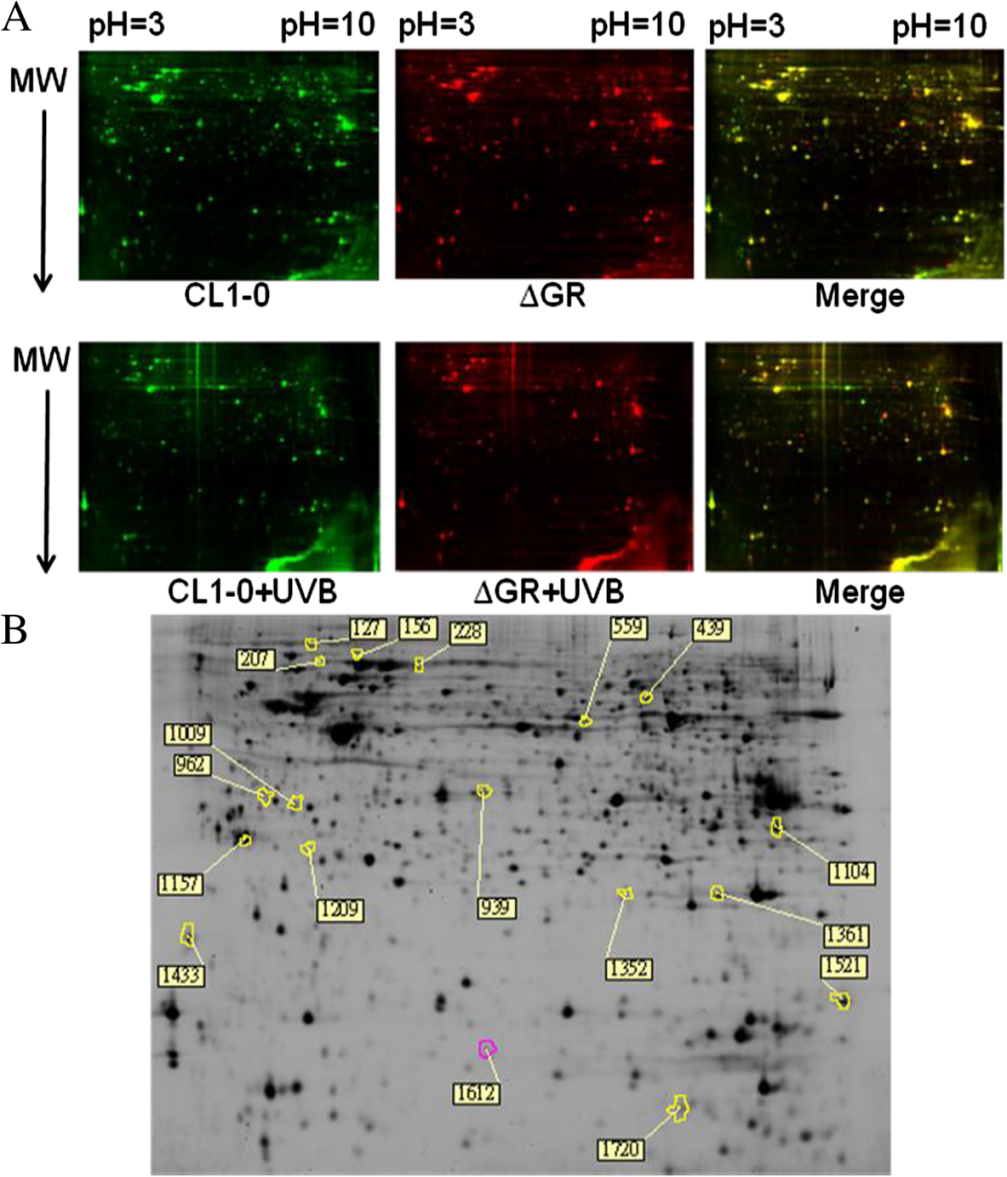
**2D-DIGE analysis of UVB-dependent differentially expressed proteins in CL1-0 and CL1-0ΔGR cells.** UVB-irradiated CL1-0 and CL1-0∆GR cells were lysed and arranged for a triplicate 2D-DIGE experiment. Protein samples (150 μg each) were labeled with Cy-dyes and separated using 24 cm, pH 3–10 non-linear IPG strips. 2D-DIGE images of the protein samples from pooled cell lysates and UVB-irradiated CL1-0 and CL1-0∆GR cells at 45 mJ/cm^2^ were shown as well as overlaid pseudo-colored image processed with ImageQuant Tool (GE Healthcare). Green color spots represented proteins of CL1-0 cells and CL1-0 cells irradiated with UVB. Red color spots represented proteins of ∆GR cells and ∆GR cells irradiated with UVB. The merge was shown as yellow color **(A)**. DeCyder software was further performed to detect and calculated differentially expressed proteins. The differentially expressed identified protein features are annotated with spot numbers **(B)**.

**Figure 5:**
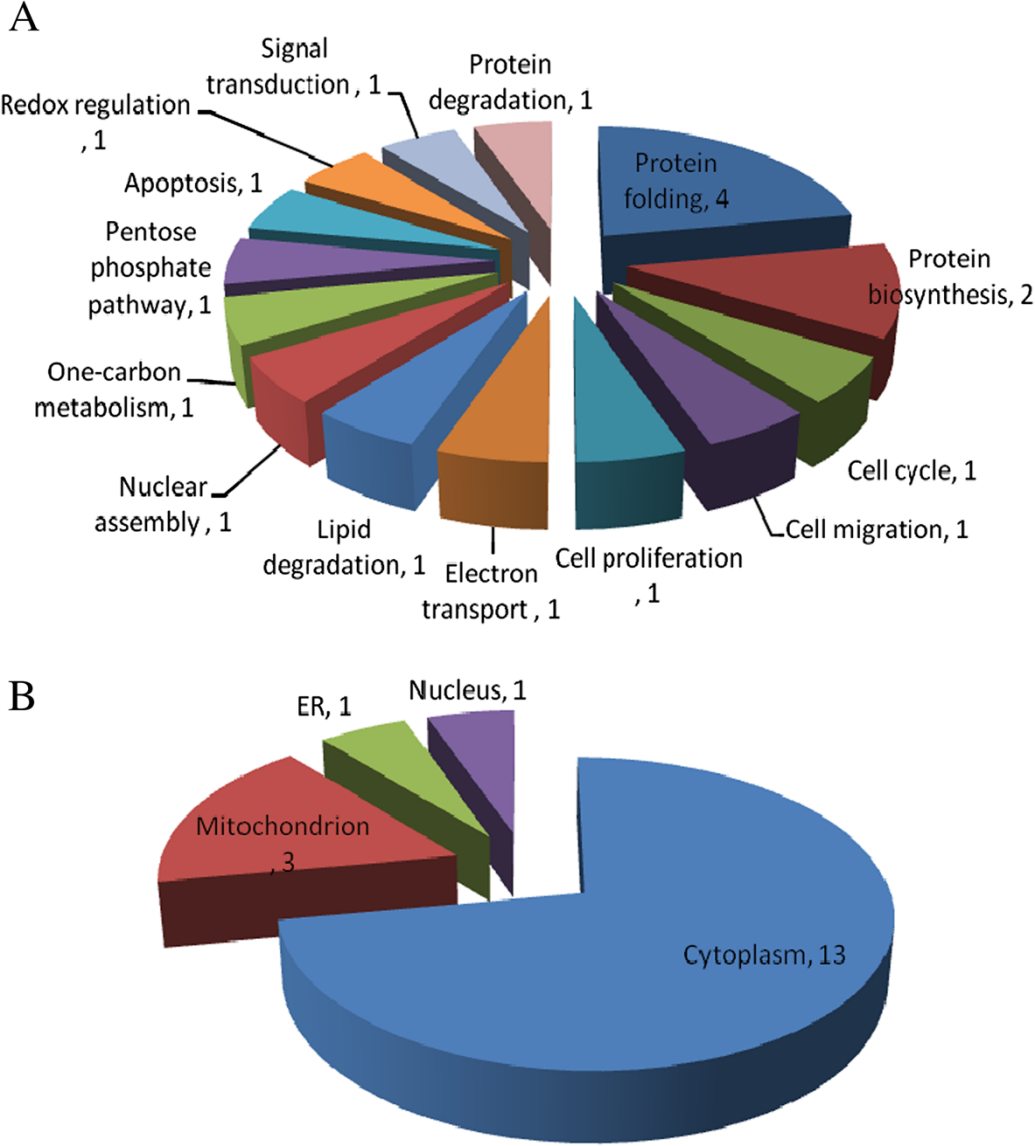
**Percentage of identified differentially expressed proteins between CL1-0 and CL1-0ΔGR in response to UVB-irradiation according to their biological functions (A) and subcellular locations (B).** The classification of the biological functions and sub-cellular locations of these identified proteins are based on a Swiss-Prot search and KEGG pathway analysis.

We previously showed that 45 mJ/cm^2^ UVB induced a significant loss of cell viability in both CL1-0 and CL1-0ΔGR cells as well as generated a maximal ROS production at 2 h post-UVB irradiation. In order to globally analyze the roles of GR in response to UVB irradiation and associated proteome alterations, CL1-0 and CL1-0ΔGR cells obtained at 2 h after 45 mJ/cm^2^ UVB irradiation or unirradiated were analyzed by a biological triplicated 2D-DIGE to examine the protein expression changes. The analysis revealed 1902 protein spots were detected and 18 protein features that displayed significantly differential expression in CL1-0ΔGR cells (≧1.3-fold; *P* < 0.05) but not in CL1-0 cells (≦1.2-fold; *P* < 0.05) were subsequently identified by MALDI-TOF MS (Table 1 and Figure 4). Using functional information from the KEGG and Swiss-Prot pathway databases, numerous biological functions were ascribed to the identified proteins with possible roles in GR depletion-induced proteome alterations against UVB irradiation. Figure 5 compares the expression profiles of the differentially expressed proteins. Proteins known to regulate protein folding, metabolism and protein biosynthesis were found to be altered abundances in between CL1-0 and CL1-0ΔGR cells. Almost two-third of the differentially expressed proteins identified were cytosolic proteins (Figure 5 and Table 1).

**Table 1:** List of identified differentially expressed proteins between CL1-0 and CL1-0ΔGR in response to UVB-irradiation

**Spot no.**	**Swiss-prot no.**	**Protein name**	**pI**	**MW**	**No. matched peptides**	**Cov. (%)**	**Score**^**a**^	**CL1 + UVB/CL1**^**b**^	**∆GR + UVB/∆GR**^**b**^	**Functional classification**	**Subcellular location**
1157	P31946	14-3-3 protein beta/alpha	4.76	28181	16/46	43%	108/56	-1.2	-1.3	Signal transduction	Cytoplasm
962	P29692	Elongation factor 1-delta	4.9	31219	9/40	32%	77/56	1.11	1.46	Protein biosynthesis	Cytoplasm
1720	P41567	Eukaryotic translation initiation factor 1	6.89	12841	4/13	42%	63/56	-1.09	2.24	Protein biosynthesis	Cytoplasm
439	Q16658	Fascin	6.84	55134	8/24	18%	74/56	1.14	1.49	Cell migration	Cytoplasm
156	P11142	Heat shock cognate 71 kDa protein/HSC70	5.37	71086	10/30	19%	76/56	-1.06	-1.3	Protein folding	Cytoplasm
127	P08238	Heat shock protein HSP 90-beta	4.97	83560	9/22	16%	68/56	-1.15	-1.34	Protein folding	Cytoplasm
207	P20700	Lamin-B1	5.11	66658	12/41	20%	74/56	-1.2	-1.63	Nuclear assembly	Nucleus
1009	Q15691	Microtubule-associated protein RP/EB family member 1	5.02	30154	7/24	26%	75/56	1.08	1.38	Cell cycle	Cytoplasm
228	P38646	Mortalin/GRP75/Stress-70 protein, mitochondrial	5.87	73925	12/49	24%	71/56	1.06	1.61	Protein folding	Mitochondrion
1433	Q9UI09	NADH dehydrogenase [ubiquinone] 1 alpha subcomplex subunit 12	9.63	17104	5/27	19%	68/56	-1.02	-1.34	Electron transport	Mitochondrion
1521	P23284	Peptidyl-prolyl cis-trans isomerase B	9.42	23786	10/48	46%	87/56	1.05	1.72	Protein folding	ER
1361	Q06830	Peroxiredoxin-1	8.27	22328	8/40	32%	81/56	1.13	1.54	Redox regulation	Cytoplasm
1352	Q15102	Platelet-activating factor acetylhydrolase IB subunit gamma	6.33	25834	9/39	35%	101/56	1.09	1.34	Lipid degradation	Cytoplasm
559	P31153	S-adenosylmethionine synthase isoform type-2	6.02	43981	9/32	28%	85/56	1.14	1.46	One-carbon metabolism	Cytoplasm
939	P37837	Transaldolase	6.36	37690	17/57	41%	131/56	-1.12	-1.6	Pentose phosphate pathway	Cytoplasm
1209	O43399	Tumor protein D54	5.26	22282	8/32	48%	90/56	1.12	1.34	Cell proliferation	Cytoplasm
1612	P61088	Ubiquitin-conjugating enzyme E2 N	6.13	17185	6/33	35%	64/56	1.19	1.87	Protein degradation	Cytoplasm
1104	P21796	Voltage-dependent anion-selective channel protein 1 (VDAC-1)	8.62	30870	17/57	81%	201/56	-1.08	1.3	Apoptosis	Mitochondrion

^a^Protein score is -10*Log(P), where P is the probability that the observed match is a random event. Protein scores greater than 56 are significant (p < 0.05) in current searches. ^b^Average ratio of differential expression (p < 0.05) between CL1-0 irradiated with/without UVB and CL1-0ΔGR irradiated with/without UVB. The ratios were calculated from triplicate gels.

**Figure 6:**
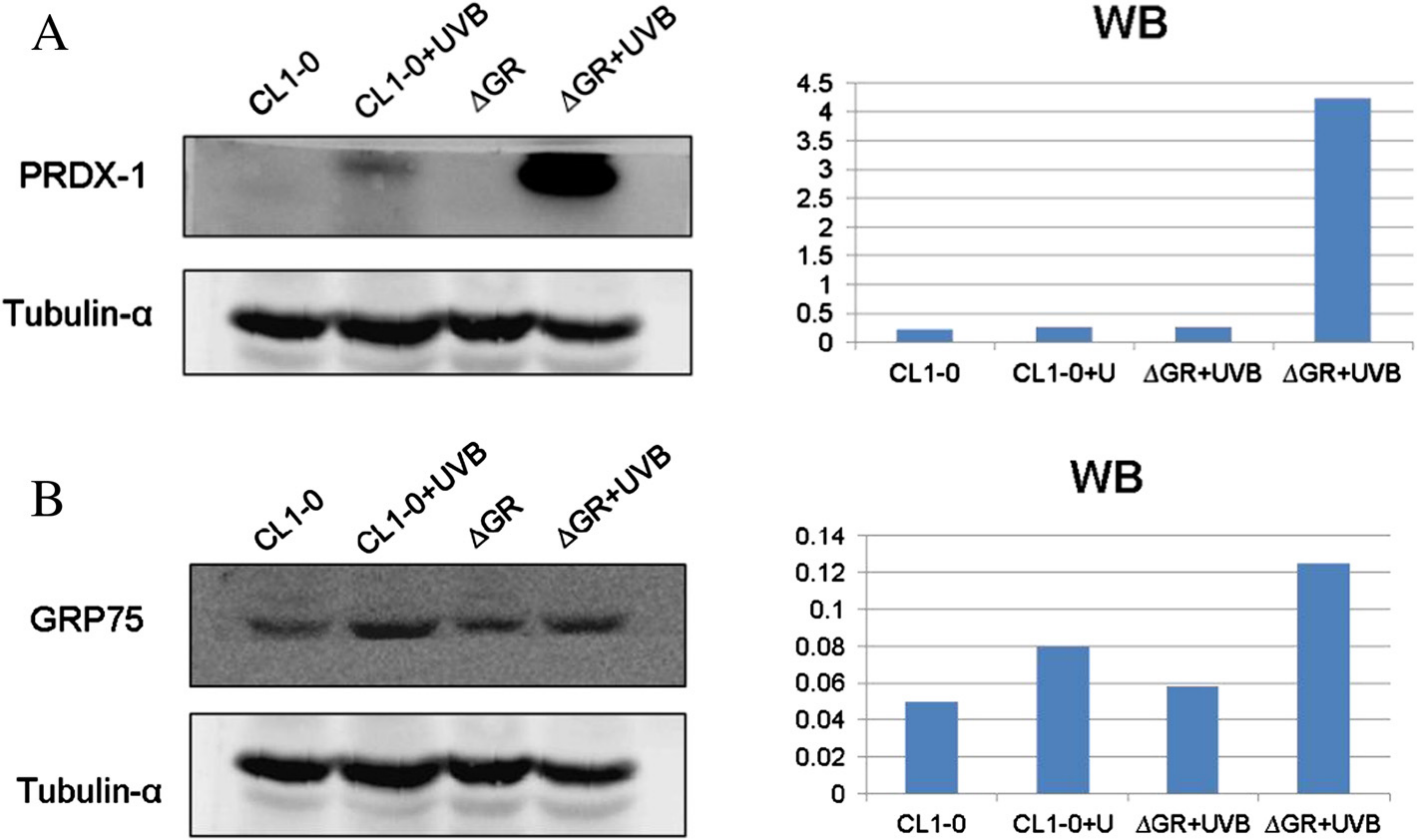
**Representative immunoblot validation of (A) Peroxiredoxin-1 (B) GRP75 levels in CL1-0 cells and CL1-0ΔGR cells in response to UVB-irradiation.** The levels of identified protein, **(A)** peroxiredoxin-1 and **(B)** GRP75, in CL1-0 and CL1-0∆GR cells irradiated with 45 mJ/cm^2^ UVB or sham exposure were confirmed by immunoblot. Beta-tubulin was used as loading control in this study (left panels) and the normalizing result between peroxiredoxin-1 / GRP75 and beta-tubulin controls were listed in the right panels.

To further verify the up-regulation or down-regulation of the identified proteins, we performed immunoblotting analysis of proteins regulated by the depletion of GR and compared it with proteins in control CL1-0 cells in response to UVB irradiation. We used specific antibodies against peroxiredoxin-1 and Glucose Regulated Protein 75 (GRP75). In general, there was a good correlation between changes observed in the 2D-DIGE analysis and in immunoblotting analysis (Figure 6). The relative expression level of beta-tubulin normalized peroxiredoxin-1 and GRP75 between UVB-irradiated CL1-0ΔGR / CL1-0ΔGR and UVB-irradiated CL1-0/CL1-0 is up-regulated for 12.46-fold and 1.35-fold in immunblotting analysis, respectively. This ratio showed the similar trend to 2D-DIGE analysis with 1.36-fold and 1.51-fold up-regulated in UVB-irradiated CL1-0ΔGR/CL1-0ΔGR and UVB-irradiated CL1-0/CL1-0 cells (Figure 6).

## Discussion

Our experimental design in performing lysine-based proteomic analysis is to monitor the role of GR in response to UVB irradiation. By means of 2D-DIGE and MALDI-TOF MS, 18 GR depletion-induced alterations in protein expression have been identified in CL1-0 cells. The results demonstrate that this experimental design is powerful enough to identify a broad-ranging signatures of GR depletion-induced proteome alterations in response to UVB irradiation, with the altered proteins having stimulation roles in cell apoptosis, cell cycle regulation, cell migration, cell proliferation, lipid degradation, one-carbone metabolism, protein biosynthesis, protein degradation, protein folding and redox regulation. In contrast, proteins involving in electron transport, nuclear assembly, pentose phosphate pathway, protein folding and signal transduction are down-regulated (Table 1).

According to STRING protein interaction database (http://string.embl.de/) using the identified proteins as input. Using the ‘medium’ confidence level defined in the software, there is evidence that 42% of these proteins can interact with one or more of the other identified proteins. Peroxiredoxin-1 can interact with the highest number of input proteins (4 interactions) including transaldolase, HSC70, VDAC1 and HSP 90-beta. The results suggest that many of the identified proteins occur within the network regulating the protein folding and pentose phosphate pathway and peroxiredoxin 1 plays a major role in coordinating events that regulate these networks in response to GR-dependent UVB-irradiation.

**Figure 7:**
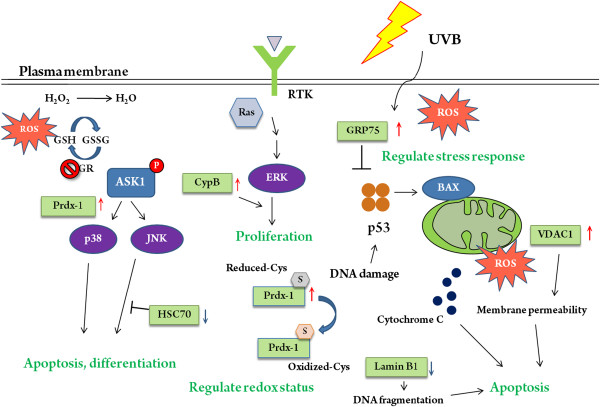
**The hypothetical model of glutathione reductase depletion-modulated protein expression in response to UVB-irradiation.** UVB-induced proteins with expression changes are shown as green rectangle. Upward arrows with red color indicate the up-regulation of the proteins. In contrast, downward arrows with blue color represent down-regulated. Detailed regulatory model of these proteins are discussed in Discussion section.

Voltage-dependent anion-selective channel protein 1 (VDAC1) located in the outer mitochondrial membrane plays crucial roles in the regulation of cell apoptosis via increasing the mitochondrial membrane permeability [29]. Our proteomic analysis indicated that UVB-induced up-regulation of VDAC1 in CL1-0ΔGR cells rather than in CL1-0 cells implying a regulatory role of GR on VDAC1 expression as well as a control role of GR on the maintaining of mitochondrial membrane permeability (Figure 7).

Lamin B1 consists of a layer of proteins located surrounding the inner nuclear membrane. During cell mitosis, lamin B1 is phosphorylated followed by reversibly disassembled into the lamin monomers. Accordingly, lamin proteins are reported to be associated with nuclear stability and chromatin organization [30,31]. In current study, UVB-induced down-regulation of lamin B1 in CL1-0ΔGR cells implying the disassembly of lamin layer was occurred as well as DNA fragmentation and cell apoptosis was initiated [32-34]. Thus, GR might play a role on the maintaining of nuclear membrane integrity and prevent UVB-induced cell apoptosis (Figure 7).

Heat shock proteins (HSP) are molecular chaperones that accelerate cell recovery from critical conditions, by reducing the concentrations of denatured or unfolded proteins [35]. In which, Hsp70 and Hsc70 are two chaperone proteins of high homology expressed under contrasting cell situations. Hsc70 is constitutively expressed, whereas Hsp70 is low-abundant in normal physiological situations and significantly induced under oxidative stress [36]. A recent report indicated that HSC70 can block the JNK/Bim signaling pathway and inhibit the translocation of Bax to mitochondria in UV-induced apoptosis. HSC70 also inhibits Bax activation by directly interacting with Bax, which avoids cytochrome c release from mitochondria and leads to anti-apoptosis in UV-irradiated cells [37]. Our data revealed that the down-regulation of HSC70 in UVB-irradiated CL1-0ΔGR cells implying GR modulated HSC70 associated chaperone functions to maintain cell viability. The same finding also showed that chaperone proteins HSP-90 was down-regulated in CL1-0ΔGR cells rather than in CL1-0 cells implying GR depletion might increase UVB-induced cell damage via deregulation of HSP-90 (Figure 7). Notably, Hsp70 and Hsp90 are not single proteins, but entire protein families. Both Hsp70 and Hsp90 are molecular chaperones that help protein folding. However, Hsp70 binds to nonnative, unstructured segments of proteins and assists in folding them. Hsp90 doesn’t bind to unfolded proteins, but to native-like proteins.

14-3-3 proteins play essential roles in the regulation of cell survival through phospho-dependent interacting to a large number of cellular proteins that are targeted by a range of protein kinases. Additionally, 14-3-3 proteins play critical roles in regulating progression of cell cycle, modulating their response to DNA damage, responding to UV-induced damages and influencing cell death/survival decisions [38]. Our result pointed out 14-3-3 proteins were down-regulated in CL1-0ΔGR cells in response to UVB-irradiation implying GR depletion might down-regulate 14-3-3 protein and increase UVB-induced cell death (Figure 7).

Cyclophilins are protein chaperones that accelerate the rate of protein folding through their peptidyl-prolyl cis-trans isomerase (PPIase) activity and catalyze the cis-trans isomerization of proline imidic peptide bonds in oligopeptides. A number of studies showed that cyclophilin B has an essential function in protecting hepatoma cells against oxidative stress through binding to CD147 and regulating the ERK pathway [39] and overexpressed cyclophilin B suppresses apoptosis associated with ROS and Ca^2+^ homeostasis under ER stress [40]. In our results, cyclophilin B was up-regulated in UVB-irradiated CL1-0ΔGR cells implying that cyclophilin B plays a role to accelerate protein folding in response to oxidative stress when GR was depleted and this process might be associated with the ERK signaling pathway to promote cell survival (Figure 7).

In conclusion, the current study used a comprehensively proteomic approach for the identification of GR-modulated protein expression in response to UVB irradiation. In addition to the expected induction of a set of folding-associated proteins in GR depleted CL1-0 cells, this study also found a number of metabolic proteins modulated in GR depleted CL1-0 cells. Further analysis demonstrated that cellular proteins involving redox-regulation were also modulated by GR-depletion in protein expression. To our knowledge, this is the first global proteomic analysis to investigate the role of GR in response to UVB irradiation in mammalian cell model.

## Abbreviations

2-DE: Two-dimensional gel electrophoresis; CCB: Colloidal coomassie blue; DIGE: Differential gel electrophoresis; GR: Glutathione reductase; MALDI-TOF MS: Matrix assisted laser desorption ionization-time of flight mass spectrometry; ROS: Reactive oxygen species; RSH: Free thiol group; VDAC1: Voltage-dependent anion-selective channel protein 1.

## Competing interests

The authors confirm that there are no conflicts of interest.

## Authors’ contributions

HCC designed the experiments and the draft manuscript writing. HCC supervised the experiments and the data analysis and finalized the manuscript. Both authors have read and approved the final manuscript.
